# Reference the seabed topographic depth observations based on the national mean dynamic topography model

**DOI:** 10.1016/j.mex.2024.102624

**Published:** 2024-02-22

**Authors:** Thanh Thach Luong, An Dinh Nguyen, Dinh Hai Nguyen, Van Hai Tran, Nhung Le, Thi Thanh Tam Le, Thi Thanh Thuy Pham, Dinh Thanh Nguyen, Thi-Nhung Do

**Affiliations:** a Hanoi University of Natural Resources and Environment, No. 41A, Phu Dien Street, Phu Dien Ward, Bac Tu Liem District, Hanoi, Viet Nam; b Surveying and Aerial Mapping Co., Ltd, Viet Nam; c Vietnam People Naval Hydrographic and Oceanographic Department, Viet Nam; d The GFZ German Research Centre for Geosciences in Potsdam, Federal Republic of Germany; e Hanoi University of Mining and Geology, No.18 Vien Street, Duc Thang Ward, Bac Tu Liem District, Hanoi, Viet Nam; f Vietnam Association of Geodesy, Cartography, and Remote Sensing, Viet Nam; g VNU University of Science, Vietnam National University, Hanoi, 334 Nguyen Trai, Thanh Xuan, Hanoi, Viet Nam

**Keywords:** Vietnam mean sea surface, Vietnam seabed topography, National mean dynamic topography model, Water depth measurement, MDTVN22, Mean dynamic topography Vietnam 22 (MDTVN22)

## Abstract

The mean sea surface in different regions is non-equipotential, rendering Vietnam's traditional approach, which relies on the Hon-Dau tide gauge station as a reference, not yet scientifically invalid. To overcome this, our study utilized the Vietnam national mean dynamic topography model (MDTVN22) for depth observations, particularly in the Gulf of Tonkin. Covering 3430 monitoring sites in Hai Phong and 813 sites in Quang Ninh, our experiments highlighted a 5 to 6 mm difference between the mean sea surface and MDTVN22 references.

• Our research establishes a resilient methodology, integrating shore tide gauge station data and the MDTVN22 model, aimed at enhancing precision in depth observations.
• Validation experiments in Hai Phong demonstrate a minimal discrepancy of ±0.006 m between measurements obtained from the traditional mean sea surface and the MDTVN22 model.
• These findings underscore the significance of adopting the MDTVN22 model for improved accuracy in assessing Vietnam's seabed topography.

## Graphical abstract

**Figure:**
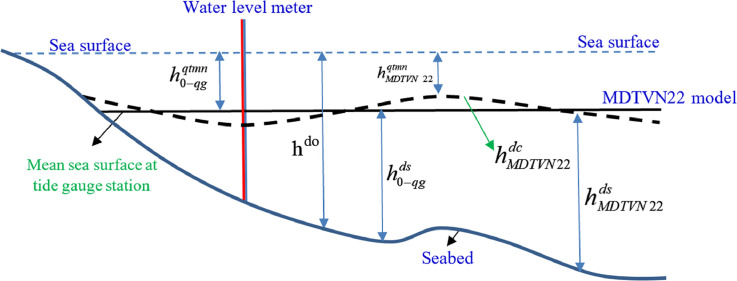

Specifications table

**Table:** 

Subject area:	Earth and Planetary Sciences
More specific subject area:	Seabed topographic depth observations
Name of your method:	Mean dynamic topography Vietnam 22 (MDTVN22)
Name and reference of original method:	*n/a*
Resource availability:	*n/a*

**Method details**

## Introduction

According to the mathematical basis [1,2], the seabed topographic map is shown as the seaward extension of the national topographic map on land. Therefore, the seabed topographic map and the national topographic map on land constitute a consistent system both in the reference system, coordinate system and elevation system, not only in content but also in the representation of geographic features [3]. The seabed topographic map plays a critical role in the construction and socio-economic development of the sea, as a basis for designing and constructing coastal constructions on islands and archipelagoes, developing fisheries, planning sea transport routes, researching the environment, and exploiting gas. The most important geographic feature on the seabed topographic map is the seabed topographic depth. Seabed topographic maps are used as a database of marine geographic information in the surveying and mapping industry. In contrast, seabed topographic depth is an important object to refer to in marine gravity measurements.

The seabed topographic depth is referred to the mean sea surface at the coastal tide gauge station [1,4]. However, because the mean sea surfaces in different areas of the national waters are not equipotential, which means they are not on the same definite surface, considering the mean sea surface at the temporary coastal tide gauge station coincides with the mean sea surface in the location of measuring seabed topographic depth offshore to refer to the results of depth measurement of Hon Dau mean sea surface has no scientific basis [5].

The mean dynamic topography (MDT) model is a constant, fixed component of the dynamic ocean topographic surface and can be considered the global mean topographic surface of ocean circulation [6]. Knowledge of MDT is necessary for Oceanography since it provides valuable information about ocean surface flows and is also essential for the Geodetic industry. It provides the difference between the mean sea surface and the geoid surface on the sea, serving to build the national elevation system that satisfies the criterion “ensuring the compatibility of geographic information, different data systems at the national and global scope” [7,8]. Hence, many countries worldwide are currently constructing their own mean sea level (MSL) datum [9], [10], [11]. In a study [12], a comparison was made between the geodetic method and oceanography to estimate the MSL for the purpose of unifying height data in Australia based on 32 tidal gauge stations around the Australian coast. The results showed that the maximum difference in MSL estimated from the surveying method and oceanography was greater than 150 mm at some tidal stations. In another study [13], a new elliptic equation was proposed to determine MSL based on a partial differential equation of factors such as temperature, surface level, and water depth. This equation reflects the relationship between sea surface height (SSH) and geoid height. In addition, using MSL in combination with GNSS data and quasigeoid models to determine blunders and systematic errors in the coastal elevation network on the northeast coast of Australia [14]. In Ref. [15], a method is proposed to improve the accuracy of the coastal MDT by using a combination of satellite altimeter data and new elevation data at 302 tidal stations in the network. SONEL network on the northeast coast of the Atlantic Ocean. The results show a mismatch between the tidal stations and the MDT around 9.0 cm. In Refs. [16], [17], [18] shows, New Zealand is established based on topographic data of the sea surface (DNSC08) and geoid model at sea. The results at 15 tidal stations show that the standard deviation between the local vertical datum (LVD) and the MDT is 1.0 cm.

To set up a mathematical basis for the marine geographic information base and refer to the seabed topographic depth observations, meanwhile create a premise to solve scientific and practical tasks relating to the seabed topographic depth, based on the international mean dynamic topography model DTU15 MDT [19], combined with mean sea surface heights at 74 tide gauge stations along the coast and on some islands of Vietnam, study [20] has built national mean sea surface (MDTVN22) in Vietnam waters. The accuracy evaluation results of the MDTVN22 model based on 23 tide gauge stations not involved in building the model reach ±7.5 cm. Compared to the criteria for determining mean sea surface at temporary tide gauge stations based on continuous sea level measurement data for 30 days and nights with the mean error at ± 3.4 cm, the MDTVN22 model was highly accurate. It can ensure technical requirements to be used as a background for marine geographic information and a reference to the topographic depth measurements. This article introduces the MDTVN22 model to refer to the data of water level observations and seabed topographic depth measurement values in establishing seabed topographic maps and thematic sea maps on Vietnam waters.

## Material and methods

### Study area and data sources

**Fig. 1:**
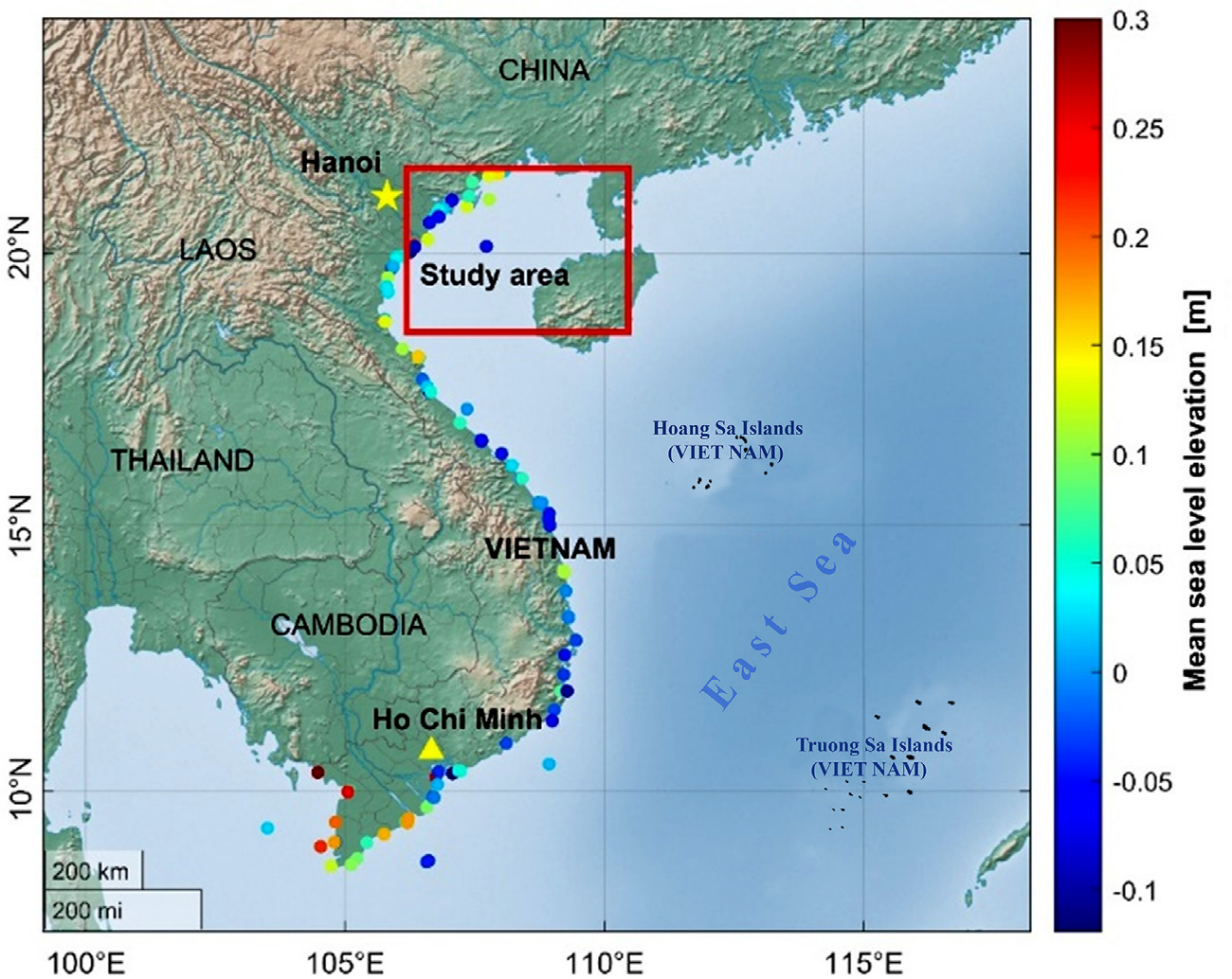
The study area with tidal gauge stations along the Vietnamese coastline.

The Gulf of Tonkin is part of the East Sea in Vietnam, with an area of about 130,000 km^2^. This sea region is the world's shallowest, most gentle, most expansive continental shelf. Its average depth is around 50 m, reaching a maximum of ∼107 m. The Gulf of Tonkin is located in geographic coordinates between 17°00′ to 21°40′ North latitude and 105°40′ to109°40′ East longitude (Fig. 1). There are more than 2370 islands here, and they are concentrated on the Northeast coast from Quang Ninh to Hai Phong. The Gulf of Tonkin has abundant natural resources such as metal ores, gems, and large oil reserves. Therefore, the Tonkin Gulf has a critical geopolitical and economic position for Vietnam.

This study used three main data sources include:

• *First data source***:** Water level observations and topographic depths were used to create the seabed topographic map at 1/25,000 scale of the Hon Long Chau Tay area of the Division of Surveying, Sea Mapping and Marine Research. The characteristics of the measuring area are described as follows (i) The sea area of Hai Phong, Vietnam (measuring location); (ii) The measured area is about 900 km^2^, of which the length is 27.7 km and the width is 29.7 km (measuring area); (iii) The tide gauge station is located at the wharf of the Border Guard station on Long Chau Island with the coordinates $\varphi =20^{o}37^{\prime }27\prime \prime N,\;\lambda =107^{o}09^{\prime }22\prime \prime E$, sea surface height *h_MBTB_ = 0.06* *m*. Point data measured in days such as 922 points (December 5, 2018 from 13:18 p.m to 23:59 p.m), 2027 points (December 13, 2018 from 00:00 a.m to 23:59 p.m), and 470 points (December 14, 2018 from 00:00 a.m to 09:48 p.m). Water level observations and seabed topographic depths observations are summarized in Appendix 1**.**
• *Second data source***:** Topographic map piece of the sea floor F-48-84-C, scale 1:50,000 in the Quang Ninh Sea area, produced by the centre for Marine Map Survey, Vietnam General Department of Sea and Islands in 2003. The characteristics of the measuring area are described as follows: (i) From 20°30′-20°45′ North latitude and 107°30′-107°45′ East longitude (Geographical location); (ii) Vietnam national coordinate system VN2000, projection zone 6°, UTM projection, meridian axis $AptCommandmathcall_{0}=105^{0}00^{\prime }00\prime \prime$; (iii) Vietnam National Elevation System Hai Phong 1972; (iv) The echo-sounding apparatus RAY THEO 719 with one frequency. Positioning by Beacon station static locator, BEACON DMS 212. The positioning software is HYDRO Nav Ver 6.07. Data of seabed topographic depths on map piece F-48-84-C are summarized in Appendix 2**.**
• *Third data source*: National mean sea surface (MDTVN22) in Vietnam waters refers to seabed topographic depths. The mean sea surface height at tide gauge stations is as follows (i) Station Ngoc Vung, sea surface height is *h_MBTB_= 0.128m*; (ii) Station Co To, sea surface height is *h_MBTB_= 0.113m*; and (iii) Station Bach Long Vi, sea surface height is *h_MBTB_ = −0.06m*.

### Methodology

According to current technical regulations, the seabed topographic map's depth is referred to on the Hon Dau mean sea surface, based on the determination of Hai Phong-1972 national elevation for the tidal gauge station of the measuring area by geometric levelling method or GNSS [1]. Only one tide gauge station will be used within a small monitoring area with consistent tidal characteristics. The increase in tidal gauge stations depends on the tidal characteristics in the measuring area [21]. This provision has two purposes: (i) Refer the seabed topographic depth to the mean sea surface based on the water level observation data of the temporary coastal tide gauge station; (ii) Connecting the seabed topographic depth to the national elevation system of Hai Phong-1972.

Nearly 30 years after establishing the seabed topographic map, the ratio and thematic maps for economic and social development in Vietnam comply with the above provisions. However, this provision has many limitations when considering the entire measuring area on the same plane passing through the mean sea surface at the tide gauge station. The mean sea surface in different sea areas is different [22,23]. On the other hand, when the adjacent measuring areas have different tide gauge stations, the depth at the boundary area is "matched" based on the subjective judgment of the industrial editors. This leads to many errors in the depth measured in the adjacent places, especially the ones with two areas of different tidal characteristics. The advantage of referencing depth based on the sea surface models is to accurately determine the height of the referenced sea level at each depth measuring location and not adjacent to the measuring areas with different tidal characteristics, meanwhile creating favorable conditions for solving problems using seabed topographic depth measurement data for different purposes.

**Fig. 2:**
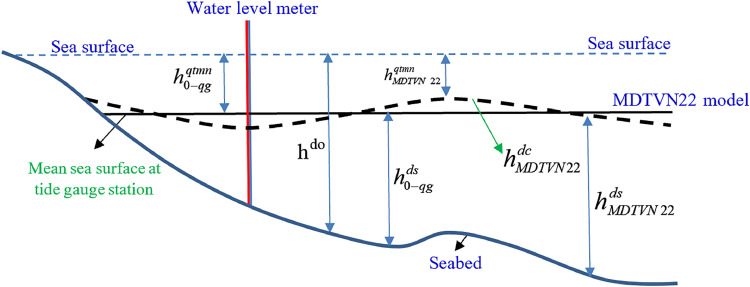
Referring water level observations and seabed topographic depths based on the national MDTVN22 model.

The problem of referring to seabed topographic measurement and the mean sea level observations at a tide gauge station has been published in many documents [1,5]. The authors will not repeat that simple algorithm here. Instead, the following content will present the method of referring water level observations and seabed topographic depths based on the national MBTBN2022 model. Fig. 2 illustrates the principle of referring observations of seawater levels and seabed depths based on the national MDTVN22 model.

• *Referring seawater level observations using the MDTVN22 model*

The equation calculates water level observations based on the MDTVN22 model.

(1) $$h_{MDTVN22}^{qtmn}=h_{0\_qg}^{qtmn}-\;h_{MDTVN22}^{dc}$$

in which: $h_{0\_qg}^{qtmn}\;$is the water level observations based on the mean sea surface at the tide gauge station; $h_{MDTVN22\;}^{qtmn}$is the water level observations based on the MDTVN22 model; $h_{MDTVN22}^{dc}$ is the height of the MDTVN22 model. To calculate the value in column (8) of Appendix 3, we used Eq. (1):

• *Referring seabed depths observations using the MDTVN22 model*

Seabed topographic depths based on the MDTVN22 model are calculated by the equation as follows.

(2) $$h_{MDTVN22}^{ds}=h_{MDTVN22}^{qtmn}-\;h^{ds}$$

in which: $h_{MDTVN22}^{ds}$ is seabed topographic depth based on the MDTVN22 model; $h_{MDTVN22}^{qtmn}\;$is the water level observations based on the MDTVN22 model; and $h^{ds}\;$is the height from the sea surface to the seabed. The study used formula 2 to calculate the value in column (9) Appendix 3.

Conducting experimental calculations by the method used above to refer to the water level observations is often divided into two cases: referring based on the mean sea surface height at the tide gauge station and referring based on the MDTVN22 model. Similarly, the seabed topographic depth is also referred for two cases: (1) based on the mean sea surface height at the tide gauge station and (2) based on the MDTVN22 model. The calculated results are shown in Appendix 3.

**Fig. 3:**
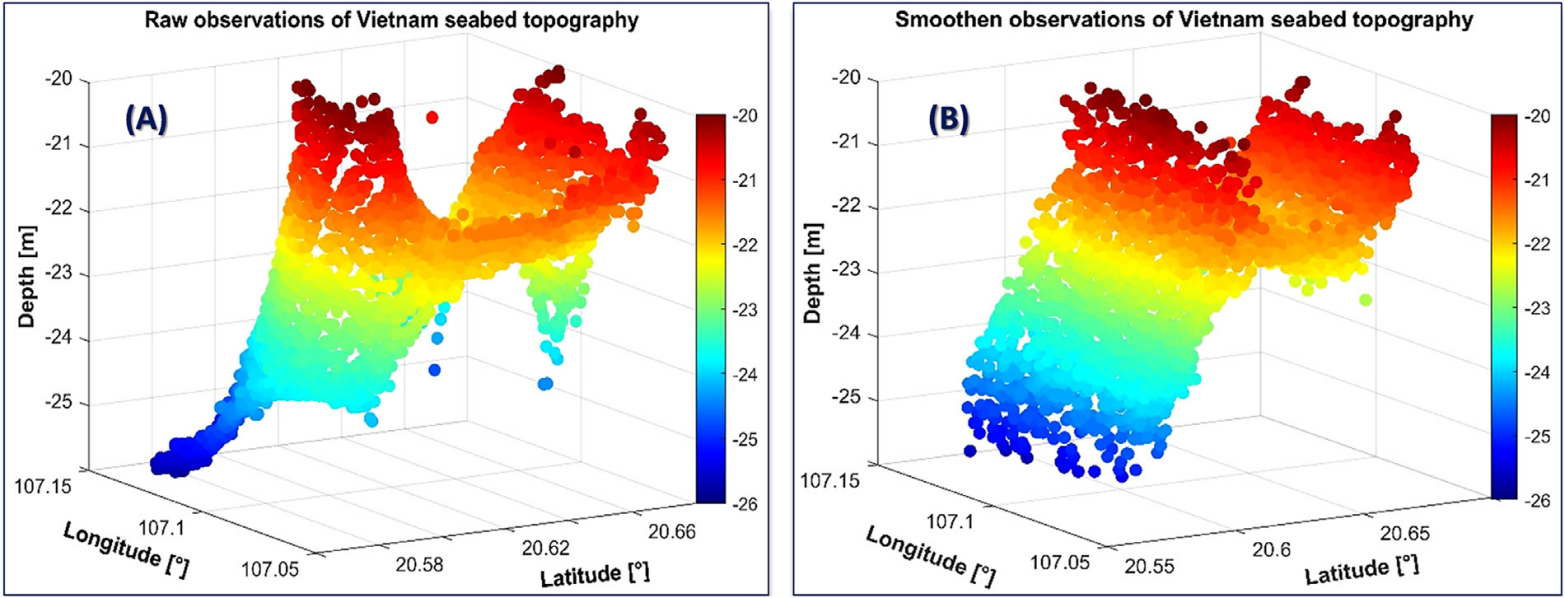
The depth observations in the study area before (A) and after (B) cleaning outliers.

We first applied the moving median algorithm to remove outliers (i.e., gross errors) in the observations (Fig. 3). Then, spline interpolations were also employed to fill out these missing values. In this way, the quality of the input data will be improved, while the number of observations will still be preserved after removing observations identified as outliers.

• *Method for referring seabed depths observations using the MDTVN22 model*

The algorithm for referring to the seabed topographic depth based on the MDTVN22 model still relies on the observation data of water level and mean sea surface height at the tide gauge station. Many areas already have seabed topographic maps (seabed topographic depth refers to the mean sea surface height at tide gauge stations). However, there is no information on the tide gauge station's water level observations, coordinates, or heights. To solve this problem, we chose the coordinates and heights at a tide gauge station in the database close to the area of the seabed topographic map piece and referred to the depth observations based on the MDTVN22 model. The procedure for referring the depth observations of the seabed topographic map based on the MDTVN22 model is as follows:

The difference between the height of the tide gauge station and the height of the MDTVN22 model is computed as follows:

(3) $$\Delta h=h_{0\_\text{qg}}^{dc}-h_{\text{MDTVN}22}^{dc}$$

in which: $h_{0\_\text{qg}}^{dc}$ is the mean sea surface height in the national elevation system; $h_{\text{MDTVN}22}^{dc}$ is the model height at the seabed topographic depth measured site. The further results are shown in column (7) in Appendix 4.

The depth of the seabed topographic map based on the MDTVN22 model is as follows.

(4) $$H_{\text{MDTVN}22}^{ds}=h_{0\_\text{qg}}^{ds}-\;\Delta h$$

in which: $h_{\text{MDTVN}22}^{ds}$ is the depth of the seabed topographic map using the MDTVN22 model; $h_{0\_\text{qg}}^{ds}$ is the depth of the seabed topographic map The specific results are shown in column (8), Appendix 3.

From the results in Appendix 3, we determine systematic errors:

(5) $$A=\sum_{i=1}^{3430}d_{i}^{\mathrm{tb}}\;=\;118.165m,B=\;\sum_{i=1}^{3430}\left|d_{i}^{\mathrm{tb}}\right|=118.165m$$

in which: |A|=118.165>0.25×B=29.541.

Eliminate systematic errors by the Bessel method (i) Calculate the correction number $\delta^{tb}\;$by the Eq. (6), and (ii) Eliminate the systematic errors from the difference d_i_ by the Eq. (7).

(6) $$\delta^{tb}=\frac{\left[d^{tb}\right]}{n}=0.034m$$

(7) $$\varepsilon_{i}^{tb}=d_{i}^{tb}-\;\delta^{tb}$$

The difference $\varepsilon_{i}^{tb}$ does not contain system errors. Calculate mean square error by the Eq. (8).

(8) $$m^{tb}=\pm \sqrt{\frac{\left[\varepsilon \varepsilon \right]}{2\left(n-1\right)}}=\pm \sqrt{\frac{0.227}{2\;\times \;3429}}=\pm 0.006m$$

Further investigations on the sea area of Quang Ninh (corresponding to a map piece F-84-48-C, with a scale of 1:50,000), the results are shown in Appendix 4.

• *Method for referring seawater level observations using the MDTVN22 model*

As shown in Appendix 4, we corrected systematic errors in the seawater level observations using the Bessel method. The experimental results on 813 monitoring sites in Quang Ninh determine that the systematic error is ±5 mm (please see Eq. (10)).

(9) $$A=\sum_{i=1}^{813}d_{i}^{\text{hs}}=13.519m,B=\sum_{i=1}^{813}\left|d_{i}^{\text{hs}}\right|=13.525\;m$$

in which: |A|=13.519>0.25×B=3.381.

The above two measurement ranges contain systematic errors.

(10) $$m^{bd}=\pm \sqrt{\frac{\left[\varepsilon \varepsilon \right]}{2\left(n-1\right)}}=\pm \sqrt{\frac{0.043}{2\;\times \;812}}=\pm 0.005\;m$$

We choose two other stations in Co To (the mean sea surface height *h_mean_= 0.113* *m*) and Bach Long Vi (the mean sea surface height *h_mean_= −0.06* *m*). At the Co To tide gauge station:

(11) $$m^{bd}=\pm \sqrt{\frac{\left[\varepsilon \varepsilon \right]}{2\left(n-1\right)}}=\pm \sqrt{\frac{0.005}{2\;\times \;812}}=\pm 0.002\;m$$

At the Bach-Long-Vi tide gauge station:

(12) $$m^{bd}=\pm \sqrt{\frac{\left[\varepsilon \varepsilon \right]}{2\left(n-1\right)}}=\pm \sqrt{\frac{0.043}{2\;\times \;812}}=\pm 0.005\;m$$

The results of assessing the accuracy of the three tide gauge stations mentioned above show that the results referring to the depth of the seabed topographic map do not be affected considerably by the coastal tide gauge station selection.

**Fig. 4:**
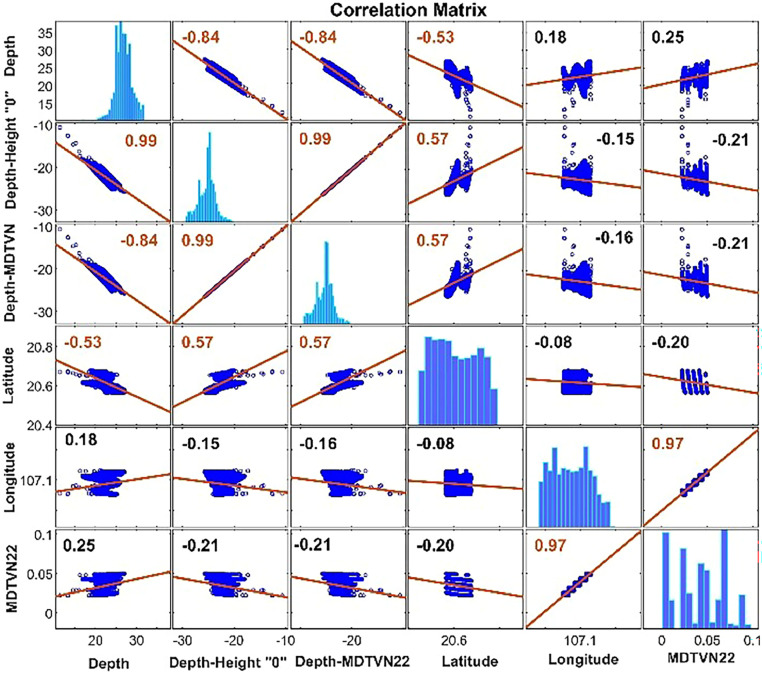
Correlation matrix between the observations and the MTDVN22 model.

The systematic errors also demonstrate that using the mean sea surface as references of seabed observations does not guarantee reliability. Furthermore, as indicated in Fig. 4, the observations and the MTDVN22 model also show a higher correlation, with ∼0.99, while the correlation between observations and the mean sea surface (i.e., Depth-Height “0”) is only at 0.84 (Fig. 4). These findings advocate that the MTDVN22 model is the best-fitted surface for the seabed topography.

**Fig. 5:**
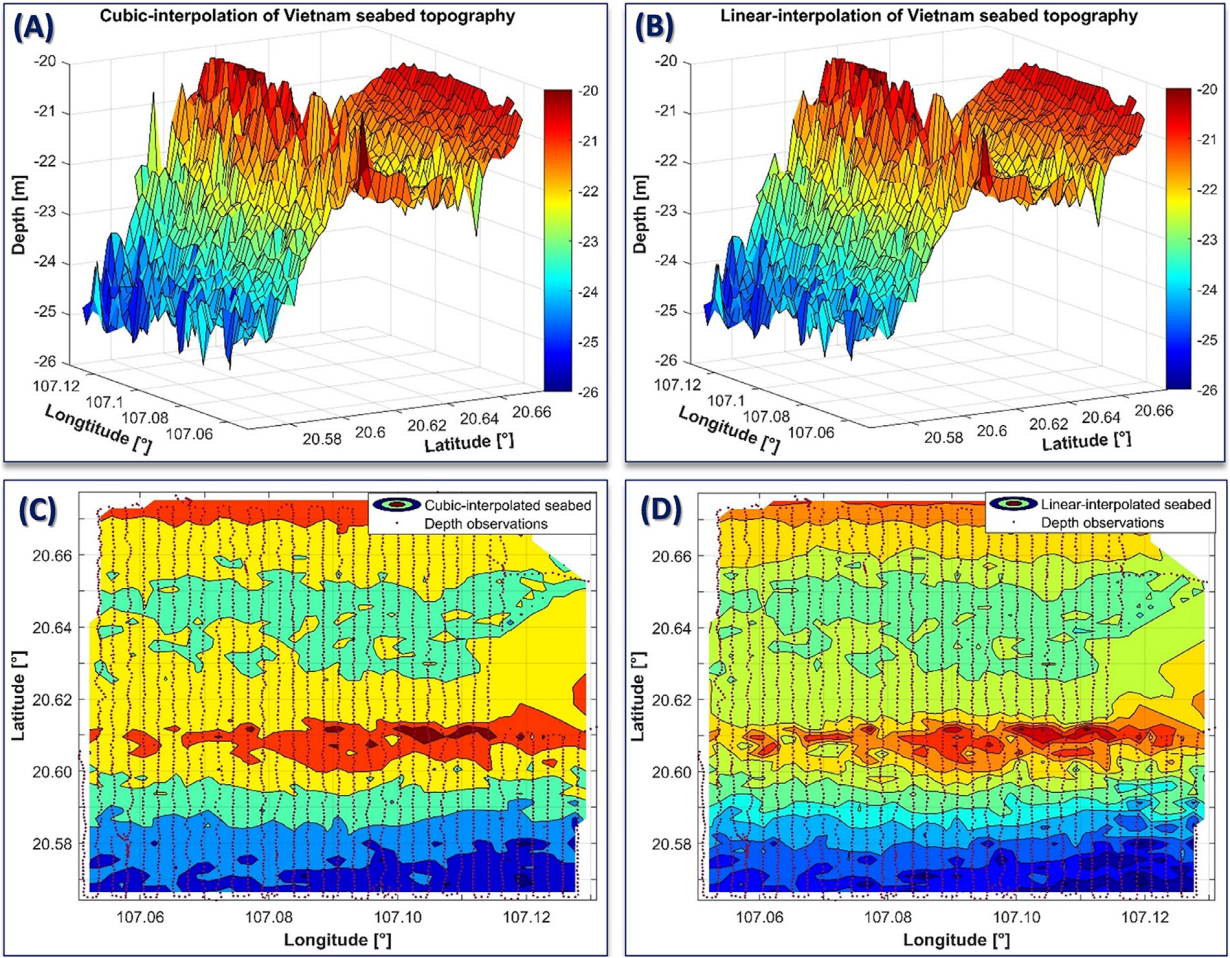
The 3D and 2D models of the seabed topography in the experimental region using the cubic (A) and (C) and linear (B) and (D) interpolation algorithms.

Fig. 5 shows further applications of our methodology to build the 2D and 3D digital models for the seabed topography, in which the MDTVN22 model was used as a reference to convert the depth observations (Fig. 5). The cubic and linear interpolation algorithms were applied to build these digital models. There is a high agreement between the two interpolation methods. Using these digital models, we can extract any seabed site on the MDTVN22 reference in the experimental region. Please note that we experimented in the sea regions of Hai Phong and Quang Ninh, Vietnam. However, the methodology in this study can be applied to different regions, where the MDTVN22 model covers. In that case, the accuracy of the seabed topographic models and maps will depend on the quality of the input data.

## Conclusions

Our study proves that using the MDTVN22 models as a reference surface of the seabed depth measurements, is accurate and certain in the methodology. The experimental results also indicate that the MDTVN22 models can be used as a mathematical basis datum for mapping the seabed topography in Vietnam.

Moreover, once the lowest sea surface model is built (known as LAT_VN) and combined with the MDTVN22 model, it will be more practical to convert the seabed topographic depth at each location from one reference model to another. Thus, the MDTVN22 model and the LAT_VN model can be used to convert the nautical chart depth to the seabed topographic depth to establish a seabed topographic map covering the entire sea region of Vietnam.

The disadvantage of the presented method is that it still has to rely on water level monitoring data at the tidal stations along the coastline. Therefore, further investigations should be carried out for a better solution.

## CRediT authorship contribution statement

**Thanh Thach Luong:** Conceptualization, Validation, Writing – review & editing, Formal analysis, Visualization, Project administration. **An Dinh Nguyen:** Validation, Formal analysis, Visualization. **Dinh Hai Nguyen:** Validation, Formal analysis, Visualization. **Van Hai Tran:** Validation, Formal analysis, Visualization. **Nhung Le:** Writing – review & editing, Formal analysis, Visualization, Project administration. **Thi Thanh Tam Le:** Validation, Formal analysis, Visualization. **Thi Thanh Thuy Pham:** Validation, Writing – review & editing, Formal analysis, Visualization, Project administration. **Dinh Thanh Nguyen:** Validation, Formal analysis, Visualization. **Thi-Nhung Do:** Conceptualization, Writing – review & editing, Formal analysis, Visualization, Project administration.

## Declaration of competing interest

The authors declare that they have no known competing financial interests or personal relationships that could have appeared to influence the work reported in this paper.

## Data Availability

Data will be made available on request.

## Appendix Supplementary materials

Image, application 1
