# Supplementary material for: Reference the seabed topographic depth observations based on the national mean dynamic topography model
Source: MethodsX. 2024 Feb 22;12:102624. doi: 10.1016/j.mex.2024.102624 (PMC10907200; doi:10.1016/j.mex.2024.102624)
Supplement: Supplementary file 1 [file mmc1.docx]

**Supplementary material *and/or* additional information [OPTIONAL]**

**Appendix**

**Appendix 1.** Water level observations and seabed topographic depths

| **No.** | **Time** | **Latitude**  () | **Longitude**  () | **Depth of measurement (m)** | **Water level observations based on mean sea level at tide gauge stations (m)** |
| --- | --- | --- | --- | --- | --- |
| **December 5, 2018** | | | | | |
| 1 | 13:18:41 | 20 36 19.8223N | 107 03 02.4275E | 23.50 | 1.371 |
| 2 | 13:19:21 | 20 36 16.3852N | 107 03 02.0197E | 23.60 | 1.371 |
| 3 | 13:20:01 | 20 36 14.8239N | 107 03 01.7218E | 23.60 | 1.311 |
| 4 | 13:20:41 | 20 36 12.2787N | 107 03 01.9591E | 23.60 | 1.311 |
| 5 | 13:21:22 | 20 36 09.3734N | 107 03 03.0897E | 23.50 | 1.311 |
| . . . | . . . | . . . | . . . | . . . | . . . |
| 918 | 23:56:48 | 20 39 14.7259N | 107 04 11.5490E | 21.00 | -1.139 |
| 919 | 23:57:28 | 20 39 10.3997N | 107 04 11.6769E | 21.00 | -1.139 |
| 920 | 23:58:10 | 20 39 05.9103N | 107 04 11.1174E | 21.00 | -1.139 |
| 921 | 23:58:51 | 20 39 01.6561N | 107 04 11.6576E | 21.70 | -1.139 |
| 922 | 23:59:31 | 20 38 57.3832N | 107 04 10.8799E | 18.00 | -1.139 |
| **December 13, 2018** | | | | | |
| 923 | 00:00:13 | 20 38 52.8880N | 107 04 10.2343E | 21.30 | -1.129 |
| 924 | 00:00:54 | 20 38 48.5418N | 107 04 10.3888E | 21.20 | -1.129 |
| 925 | 00:01:34 | 20 38 44.1793N | 107 04 10.0024E | 21.00 | -1.129 |
| 926 | 00:02:16 | 20 38 39.8576N | 107 04 10.8305E | 20.80 | -1.129 |
| 927 | 00:02:56 | 20 38 35.5427N | 107 04 10.5038E | 20.80 | -1.129 |
| . . . | . . . | . . . | . . . | . . . | . . . |
| 1965 | 11:59:58 | 20 38 49.5766N | 107 05 27.9769E | 23.70 | 1.691 |
| 1966 | 12:00:38 | 20 38 53.2531N | 107 05 28.0361E | 23.70 | 1.691 |
| 1967 | 12:01:18 | 20 38 57.0070N | 107 05 28.2156E | 23.80 | 1.691 |
| 1968 | 12:02:03 | 20 39 01.2082N | 107 05 27.3108E | 23.80 | 1.691 |
| 1969 | 12:02:43 | 20 39 04.9548N | 107 05 27.2775E | 23.70 | 1.691 |
| . . . | . . . | . . . | . . . | . . . | . . . |
| 2946 | 23:57:10 | 20 37 23.5383N | 107 06 43.1554E | 19.30 | -1.189 |
| 2947 | 23:57:51 | 20 37 19.8183N | 107 06 42.8287E | 18.60 | -1.189 |
| 2948 | 23:58:31 | 20 37 16.0777N | 107 06 42.8559E | 17.80 | -1.189 |
| 2949 | 23:59:11 | 20 37 12.2831N | 107 06 42.7329E | 17.30 | -1.189 |
| 2950 | 23:59:52 | 20 37 08.5803N | 107 06 42.7471E | 17.00 | -1.219 |
| **December 14, 2018** | | | | | |
| 2951 | 00:00:33 | 20 37 04.8754N | 107 06 42.3860E | 16.90 | -1.219 |
| 2952 | 00:01:13 | 20 37 01.2021N | 107 06 42.4451E | 16.80 | -1.219 |
| 2953 | 00:01:53 | 20 36 57.6248N | 107 06 42.4502E | 16.80 | -1.219 |
| 2954 | 00:02:33 | 20 36 54.1238N | 107 06 42.7044E | 16.90 | -1.219 |
| 2955 | 00:03:13 | 20 36 50.7256N | 107 06 42.2257E | 17.20 | -1.219 |
| . . . | . . . | . . . | . . . | . . . | . . . |
| 3426 | 09:46:16 | 20 36 34.0724N | 107 07 41.6996E | 22.70 | 1.401 |
| 3427 | 09:46:52 | 20 36 37.9454N | 107 07 42.0602E | 21.90 | 1.401 |
| 3428 | 09:47:28 | 20 36 40.0331N | 107 07 44.9815E | 21.80 | 1.401 |
| 3429 | 09:48:04 | 20 36 42.1305N | 107 07 48.0723E | 21.50 | 1.401 |
| 3430 | 09:48:40 | 20 36 43.9672N | 107 07 51.3970E | 21.00 | 1.401 |

**Appendix 2.** Data of seabed topographic depths.

| **No.** | **Latitude**  () | **Longitude**  () | **Elevation  (m)** |
| --- | --- | --- | --- |
| **(1)** | **(2)** | **(3)** | **(4)** |
| 1 | 20 29 40.5600N | 107 30 13.3200E | -34.1 |
| 2 | 20 30 12.6000N | 107 30 18.0000E | -31.9 |
| 3 | 20 30 12.9600N | 107 30 40.3200E | -33.2 |
| 4 | 20 29 40.9200N | 107 30 45.0000E | -32.9 |
| 5 | 20 29 40.9200N | 107 31 16.3200E | -32.3 |
| . . . | . . . | . . . | . . . |
| 400 | 20 36 59.4000N | 107 41 38.4000E | -34.8 |
| 401 | 20 36 59.4000N | 107 42 09.7200E | -35.5 |
| 402 | 20 36 26.6400N | 107 42 48.9600E | -36.5 |
| 403 | 20 36 27.0000N | 107 43 20.2800E | -38.2 |
| 404 | 20 36 59.4000N | 107 42 41.0400E | -37.3 |
| . . . | . . . | . . . | . . . |
| 809 | 20 34 00.4800N | 107 32 44.5200E | -37.1 |
| 810 | 20 42 07.9200N | 107 35 24.0000E | -30.8 |
| 811 | 20 41 35.1600N | 107 32 03.1200E | -32.2 |
| 812 | 20 40 46.5600N | 107 32 53.1600E | -32.1 |
| 813 | 20 29 57.7600N | 107 33 14.7600E | -32.5 |

**Appendix 3.** Results of referring water level observations and seabed topographic depths.

| **No.** | **Latitude** | **Longitude** | **Depth of**  **Measurement (m)** | **Water level**  **observations**  **based on**  **National**  **elevation (m)** | **Height of MDTVN22 model (m)** | **Depth referred based on National elevation**  **(m)** | **Water level**  **observations**  **based on**  **MDTVN22 model (m)** | **Depth referred**  **based on**  **MDTVN22 model (m)** | **Difference**  **Δh (m)** |
| --- | --- | --- | --- | --- | --- | --- | --- | --- | --- |
| **(1)** | **(2)** | **(3)** | **(4)** | **(5)** | **(6)** | **(7)** | **(8)** | **(9)** | **(10)** |
| **December 5, 2018** | | | | | | | | | |
| 1 | 20.6055 | 107.0507 | 23.50 | 1.371 | 0.023 | -22.129 | 1.348 | -22.152 | 0.023 |
| 2 | 20.6046 | 107.0506 | 23.60 | 1.371 | 0.023 | -22.229 | 1.348 | -22.252 | 0.023 |
| 3 | 20.6041 | 107.0505 | 23.60 | 1.311 | 0.023 | -22.289 | 1.288 | -22.312 | 0.023 |
| 4 | 20.6034 | 107.0505 | 23.60 | 1.311 | 0.023 | -22.289 | 1.288 | -22.312 | 0.023 |
| 5 | 20.6026 | 107.0509 | 23.50 | 1.311 | 0.023 | -22.189 | 1.288 | -22.212 | 0.023 |
| . . . | . . . | . . . | . . . | . . . | . . . | . . . | . . . | . . . | . . . |
| 918 | 20.6541 | 107.0699 | 21.00 | -1.139 | 0.029 | -22.139 | -1.168 | -22.168 | 0.029 |
| 919 | 20.6529 | 107.0699 | 21.00 | -1.139 | 0.029 | -22.139 | -1.168 | -22.168 | 0.029 |
| 920 | 20.6516 | 107.0698 | 21.00 | -1.139 | 0.029 | -22.139 | -1.168 | -22.168 | 0.029 |
| 921 | 20.6505 | 107.0699 | 21.70 | -1.139 | 0.029 | -22.839 | -1.168 | -22.868 | 0.029 |
| 922 | 20.6493 | 107.0697 | 18.00 | -1.139 | 0.029 | -19.139 | -1.168 | -19.168 | 0.029 |
| **December 13, 2018** | | | | | | | | | |
| 923 | 20.6480 | 107.0695 | 21.30 | -1.129 | 0.029 | -22.429 | -1.158 | -22.458 | 0.029 |
| 924 | 20.6468 | 107.0696 | 21.20 | -1.129 | 0.029 | -22.329 | -1.158 | -22.358 | 0.029 |
| 925 | 20.6456 | 107.0694 | 21.00 | -1.129 | 0.029 | -22.129 | -1.158 | -22.158 | 0.029 |
| 926 | 20.6444 | 107.0697 | 20.80 | -1.129 | 0.029 | -21.929 | -1.158 | -21.958 | 0.029 |
| 927 | 20.6432 | 107.0696 | 20.80 | -1.129 | 0.029 | -21.929 | -1.158 | -21.958 | 0.029 |
| . . . | . . . | . . . | . . . | . . . | . . . | . . . | . . . | . . . | . . . |
| 1965 | 20.6471 | 107.0911 | 23.70 | 1.691 | 0.036 | -22.009 | 1.655 | -22.045 | 0.036 |
| 1966 | 20.6481 | 107.0911 | 23.70 | 1.691 | 0.036 | -22.009 | 1.655 | -22.045 | 0.036 |
| 1967 | 20.6492 | 107.0912 | 23.80 | 1.691 | 0.036 | -22.109 | 1.655 | -22.145 | 0.036 |
| 1968 | 20.6503 | 107.0909 | 23.80 | 1.691 | 0.036 | -22.109 | 1.655 | -22.145 | 0.036 |
| 1969 | 20.6514 | 107.0909 | 23.70 | 1.691 | 0.036 | -22.009 | 1.655 | -22.045 | 0.036 |
| . . . | . . . | . . . | . . . | . . . | . . . | . . . | . . . | . . . | . . . |
| 2946 | 20.6232 | 107.1120 | 19.30 | -1.189 | 0.042 | -20.489 | -1.231 | -20.531 | 0.042 |
| 2947 | 20.6222 | 107.1119 | 18.60 | -1.189 | 0.042 | -19.789 | -1.231 | -19.831 | 0.042 |
| 2948 | 20.6211 | 107.1119 | 17.80 | -1.189 | 0.042 | -18.989 | -1.231 | -19.031 | 0.042 |
| 2949 | 20.6201 | 107.1119 | 17.30 | -1.189 | 0.042 | -18.489 | -1.231 | -18.531 | 0.042 |
| 2950 | 20.6191 | 107.1119 | 17.00 | -1.219 | 0.042 | -18.219 | -1.261 | -18.261 | 0.042 |
| **December 14, 2018** | | | | | | | | | |
| 2951 | 20.6180 | 107.1118 | 16.90 | -1.219 | 0.043 | -18.119 | -1.262 | -18.162 | 0.043 |
| 2952 | 20.6170 | 107.1118 | 16.80 | -1.219 | 0.043 | -18.019 | -1.262 | -18.062 | 0.043 |
| 2953 | 20.6160 | 107.1118 | 16.80 | -1.219 | 0.043 | -18.019 | -1.262 | -18.062 | 0.043 |
| 2954 | 20.6150 | 107.1119 | 16.90 | -1.219 | 0.043 | -18.119 | -1.262 | -18.162 | 0.043 |
| 2955 | 20.6141 | 107.1117 | 17.20 | -1.219 | 0.043 | -18.419 | -1.262 | -18.462 | 0.043 |
| . . . | . . . | . . . | . . . | . . . | . . . | . . . | . . . | . . . | . . . |
| 3426 | 20.6095 | 107.1282 | 22.70 | 1.401 | 0.049 | -21.299 | 1.352 | -21.348 | 0.049 |
| 3427 | 20.6105 | 107.1284 | 21.90 | 1.401 | 0.049 | -20.499 | 1.352 | -20.548 | 0.049 |
| 3428 | 20.6111 | 107.1292 | 21.80 | 1.401 | 0.049 | -20.399 | 1.352 | -20.448 | 0.049 |
| 3429 | 20.6117 | 107.1300 | 21.50 | 1.401 | 0.049 | -20.099 | 1.352 | -20.148 | 0.049 |
| 3430 | 20.6122 | 107.1309 | 21.00 | 1.401 | 0.049 | -19.599 | 1.352 | -19.648 | 0.049 |
|  |  |  |  |  |  |  |  |  | **118.165** |

**Appendix 4.** Results of referring the depth of seabed topographic map based on MDTVN22 model of map piece F-84-48-C scale 1:50.000 in Quang Ninh Sea area.

| **No.** | **Latitude** | **Longitude** | **Elevation  (m)** | **Height of MDTVN22 model (m)** | **Differences between mean sea level and model height (m)** | **Depth of seabed topographic map based on MDTVN22 model (m)** | **Differences between the depth of seabed topographic map and depth based on model (m)** |
| --- | --- | --- | --- | --- | --- | --- | --- |
| **(1)** | **(2)** | **(3)** | **(4)** | **(5)** | **(6)** | **(7)** | **(8)** |
| 1 | 20.4946 | 107.5037 | -34.1 | 0.102 | 0.026 | -34.126 | 0.026 |
| 2 | 20.5035 | 107.5050 | -31.9 | 0.104 | 0.024 | -31.924 | 0.024 |
| 3 | 20.5036 | 107.5112 | -33.2 | 0.104 | 0.024 | -33.224 | 0.024 |
| 4 | 20.4947 | 107.5125 | -32.9 | 0.102 | 0.026 | -32.926 | 0.026 |
| 5 | 20.4947 | 107.5212 | -32.3 | 0.102 | 0.026 | -32.325 | 0.026 |
| . . . | . . . | . . . | . . . | . . . | . . . | . . . | . . . |
| 400 | 20.6165 | 107.6940 | -34.8 | 0.108 | 0.020 | -34.820 | 0.020 |
| 401 | 20.6165 | 107.7027 | -35.5 | 0.107 | 0.021 | -35.521 | 0.021 |
| 402 | 20.6074 | 107.7136 | -36.5 | 0.107 | 0.021 | -36.521 | 0.021 |
| 403 | 20.6075 | 107.7223 | -38.2 | 0.106 | 0.022 | -38.222 | 0.022 |
| 404 | 20.6165 | 107.7114 | -37.3 | 0.107 | 0.021 | -37.321 | 0.021 |
| . . . | . . . | . . . | . . . | . . . | . . . | . . . | . . . |
| 809 | 20.5668 | 107.5457 | -37.1 | 0.109 | 0.019 | -37.119 | 0.019 |
| 810 | 20.7022 | 107.5900 | -30.8 | 0.121 | 0.007 | -30.807 | 0.007 |
| 811 | 20.6931 | 107.5342 | -32.2 | 0.123 | 0.005 | -32.205 | 0.005 |
| 812 | 20.6796 | 107.5481 | -32.1 | 0.122 | 0.006 | -32.106 | 0.006 |
| 813 | 20.4991 | 107.5541 | -32.5 | 0.102 | 0.026 | -32.526 | 0.026 |
|  |  |  |  |  |  |  | **13.519** |
